# The impact of active community-based survey on dementia detection ratio in Taiwan: A cohort study with historical control

**DOI:** 10.3389/fpubh.2022.1005252

**Published:** 2023-01-06

**Authors:** Yun-Ru Lu, Tzy-Haw Wu, Yachung Jeng, Wen-Yuan Lee, Wei-Chih Hsu, Amy Ming-Fang Yen, Shin-Liang Pan, Yen-Ching Chen, Sam Li-Sheng Chen, Hsiu-Hsi Chen, Horng-Huei Liou

**Affiliations:** ^1^Department of Neurology, China Medical University Hospital, Taipei, Taiwan; ^2^Department of Internal Medicine, National Taiwan University, Taipei, Taiwan; ^3^School of Oral Hygiene, College of Oral Medicine, Taipei Medical University, Taipei, Taiwan; ^4^Department of Neurosurgey, China Medical University Hospital, Taichung, Taiwan; ^5^Department of Neurology, Shin Kong Memorial Wu Ho-Su Hospital, Taipei, Taiwan; ^6^School of Oral Hygiene, College of Oral Medicine, Taipei Medical University, Taipei, Taiwan; ^7^Department of Physical Medicine and Rehabilitation, National Taiwan University Hospital, Taipei, Taiwan; ^8^Institute of Epidemiology and Preventive Medicine, College of Public Health, National Taiwan University, Taipei, Taiwan; ^9^Department of Neurology and Pharmacology, College of Medicine, National Taiwan University Hospital, Yunlin, Taiwan

**Keywords:** dementia, early detection, prevalence, incidence, awareness

## Abstract

**Background:**

Although early dementia detection is crucial to optimize the treatment outcomes and the management of associated symptoms, the published literature is scarce regarding the effectiveness of active screening protocols in enhancing dementia awareness and increasing the rate of early detection. The present study compared the detection ratio of an active community-based survey for dementia detection with the detection ratio of passive screening during routine clinical practice. Data for passive screening were obtained from the National Health Insurance (NHI) system, which was prospectively collected during the period from 2000 to 2003.

**Design:**

A population-based cohort study with historical control.

**Setting:**

Taiwan.

**Participants:**

A total of 183 participants aged 65 years or older were involved in a community-based survey. Data from 1,921,308 subjects aged 65 years or older were retrieved from the NHI system.

**Measurements:**

An adjusted detection ratio, defined as a ratio of dementia prevalence to incidence was used.

**Results:**

The results showed that the dementia prevalence during the 2000–2003 period was 2.91% in the elderly population, compared with a prevalence of 6.59% when the active survey was conducted. The incidence of dementia in the active survey cohort was 1.83%. Overall, the dementia detection ratio was higher using active surveys [4.23, 95% confidence interval (CI): 2.68–6.69] than using passive detection (1.45, 95% CI: 1.43–1.47) for those aged 65–79 years. Similar findings were observed for those aged 80 years and older.

**Conclusion:**

The implementation of an active community-based survey led to a 3-fold increase in the detection rate of early dementia detection compared to passive screening during routine practice.

## Background

The burden of dementia has substantially increased over the recent decades [previously estimated as a 117% increase from 1990 to 2016 (1)], exerting public health and financial burden. Recent epidemiological figures demonstrated that nearly 57 million people were living with dementia in 2019, which is projected to increase to 153 million by 2050 (2). Dementia is a progressive disorder affecting mainly the elderly population, and it is characterized by impairment in various cognitive domains to the extent that affects the social and occupational functions of the affected individuals (3). The disorder can be debilitating for the affected individuals, particularly in the advanced stage, and negatively impact the quality of life (QoL) of the patients and their families (4). To date, dementia is an incurable disease with modest clinical effectiveness of disease-modifying agents in the late stages (5, 6). Therefore, early dementia detection is crucial to optimize the treatment outcomes and the management of associated symptoms; previous reports showed that early dementia detection and treatment can positively influence the natural history and the neurodegenerative process of the disease (7).

Dementia awareness plays an important role in the early diagnosis and treatment of dementia. Primary care practitioners are vital in evaluating early signs of dementia and patient referral for specialized investigations. However, passive screening of early dementia signs may lead to under-diagnosed of the affected patients. Besides, previous reports showed that passive screening can lead to a false positive and incorrect diagnosis of some psychiatric disorders with dementia (7). Therefore, several scientific bodies have advocated active case-finding in the elderly population using validated cognitive tests and functional questionnaires (8). Despite the potential advantages of active case-finding, there is a lack of population-based studies comparing the effectiveness of active case-finding with passive screening.

Developing an indicator for awareness can be useful to reflect the general awareness of dementia in a given population. The simple ratio of prevalence to incidence (defined as the detection ratio) has been useful in neuroepidemiological studies. The disease incidence rate is a fundamental measurement of disease etiology, whereas prevalence is affected by disease awareness and detection efforts. For example, the detection ratio determined by a door-to-door survey of Parkinson's disease has demonstrated that women are more likely to seek medical attention than men (9). Combining prevalence and incidence into the detection ratio may serve as a good indicator of dementia awareness healthcare quality among patients with dementia. Higher detection ratios have been attributed to earlier diagnoses due to active detection methods using community-based surveys.

The present study compared the detection ratio of an active community-based survey for dementia detection with the detection ratio of passive screening during routine clinical practice. Data for passive screening were obtained from the National Health Insurance (NHI) system and were prospectively collected during the period from 2000 to 2003.

## Methods

### Study design

The present study was a cohort study with historical control that compared the dementia detection rate between an active community-based dementia detection survey and the passive dementia detection achieved through routine clinical practice.

### Data sources

Two data sources were utilized to estimate the prevalence of dementia using different detection methods.

#### Passive survey for dementia (population-based health insurance registry)

The dementia detection rate using the passive survey method was retrieved from the NHI system in Taiwan, which was enacted in 1995. The NHI covers more than 99% of the total population (10), and ~97% of hospitals and clinics are contracted with the NHI (11). The NHI database is available for public policy use, and this dataset can be used to perform longitudinal follow-up studies on a national cohort that includes 23 million insured people. The dataset contains information on all medical services, such as ambulatory care claims, inpatient claims, and prescriptions (12). Data from the medical records of patients with dementia who visited health service locations from 2000 to 2003 were digitized and treated as a passive survey for dementia detection among the underlying Taiwanese population.

#### Active survey for dementia (community-based dementia survey)

An active survey with a three-phase design was conducted to estimate the prevalence of dementia through an active detection method. Subjects in this study were recruited from a community-based, integrated screening program conducted in 2013 in Tainan, the southern area of Taiwan. A total of 183 participants were enrolled in our investigation.

The Chinese versions of the Mini-Mental State Examination (MMSE) and the Eight-Item Informant Interview to Differentiate Aging and Dementia (AD8) questionnaire (13) were used. The MMSE is a brief mental status test, with scores ranging from 0 to 30, for which lower values indicate greater cognitive impairments. The AD8 is a brief, sensitive screening tool that reliably differentiates between dementia and non-dementia at the very mild stage. Scores on AD8 range from 0 to 8, with higher values indicating greater cognitive impairment. The three-phase study design was as follows: (1) the AD8 questionnaire was completed by participants; (2) the Chinese version of the MMSE was administered to participants by a psychologist; and (3) participants were diagnosed by neurologists using the 2011 National Institute on Aging–Alzheimer's Association (NIA-AA) guidelines regarding criteria for all-cause dementia. Six psychologists and three neurologists participated in the screening process.

Using the MMSE, a cutoff value of 21 was established for distinguishing dementia from non-dementia. A cutoff value of 2 was selected for differentiating dementia from non-dementia using AD8.

### Statistical analysis

We first estimated the age- and sex-specific prevalence rates based on the NHI data from 2000 to 2003. We also estimated the prevalence rates for dementia based on the active community-based survey to represent the prevalence associated with early detection. Using an initially dementia-free cohort from the year 2000, we estimated the incidence rate of dementia by dividing the number of cases diagnosed with dementia during a 3-year follow-up (2000–2003) by the total person-years of the study population. The age-specific incidence of dementia during 2000–2003 was calculated as the number of new cases of dementia divided by the total person-years for each 5-year age band starting at 65 years. The dementia-free person-years contributed by each individual were calculated as the time from the date of study entry study to the end of the study, the date of first dementia diagnosis, or the date of death, whichever came first. The effects of age, sex, and geographic area on dementia incidence were assessed using a multivariable Poisson regression model.

The detection ratio was estimated as the ratio of prevalence to incidence. Adjustments were made using the Bayesian method based on the premise that prevalence follows a binominal distribution and incidence follows a Poisson distribution. Using a generalized linear model framework, the relationships between the detection ratio and covariates of interest, including age, sex, and geographic area, were regressed through a logarithm link function. After assessing the interaction terms in the Bayesian regression model, a strong interaction between age and sex was noted. Therefore, models stratified by sex were also developed. To better understand the differences in detection ratios between passive and active survey methods, we also developed a Bayesian regression model that allows for the estimation of detection ratios for each survey method.

## Results

Table 1 shows the age-specific prevalence and incidence rates of dementia in Taiwan, estimated for both the health insurance registry data and the active community-based survey. The overall prevalence rate for subjects aged 65 years and older was 2.9% using data from the health insurance registry database, which was lower than the 9.29% prevalence rate estimated from the community-based survey. Data from the NIH showed that there was a trend toward increased prevalence with age, doubling for each 5-year age until 80 years, reaching approximately 20% for the oldest group (≥90 years). A similar increasing trend in prevalence was observed in the community-based survey, but only two age groups could be assessed.

**Table 1:** Age and gender specific prevalence and incidence rate of dementia.

**Gender**	**Age**	**Prevalent case**	**Total population**	**Prevalence %**	**Incident case**	**Person years**	**Incidence %**	**Detection ratio (prevalence/ incidence)**
Male	65–69	2,620	333,391	0.79	5,757	983,219	0.59	1.34
	70–74	4,247	326,658	1.3	10,349	948,879	1.09	1.19
	75–79	6,000	205,981	2.91	13,622	575,801	2.37	1.23
	80–84	6,564	94,920	6.92	11,009	248,459	4.43	1.56
	85–89	5,041	38,710	13.02	6,279	93,985	6.68	1.95
	90+	3,445	11,363	30.32	2,278	23,246	9.8	3.09
Subtotal		27,917	1,011,023	2.76	49,774	2,873,587	1.73	1.6
Female	65–69	2,850	325,651	0.88	5,803	960,760	0.6	1.47
	70–74	5,203	255,964	2.03	8,976	742,221	1.21	1.68
	75–79	7,198	172,188	4.18	11,695	481,181	2.43	1.72
	80–84	6,481	93,801	6.91	11,098	245,000	4.53	1.53
	85–89	4,101	45,555	9	7,730	109,449	7.06	1.27
	90+	2,132	17,126	12.45	4,563	33,389	13.67	0.91
Subtotal		27,965	910,285	3.07	49,865	2,571,999	1.94	1.58
All	65–69	5,470	659,042	0.83	11,560	1,943,978	0.59	1.41
	70–74	9,450	582,622	1.62	19,325	1,691,100	1.14	1.42
	75–79	13,198	378,169	3.49	25,317	1,056,982	2.4	1.45
	80–84	13,045	188,721	6.91	22,107	493,459	4.48	1.54
	85–89	9,142	84,265	10.85	14,009	203,434	6.89	1.57
	90+	5,577	28,489	19.58	7,291	56,635	12.87	1.52
Total		55,882	1,921,308	2.91	99,609	5,445,586	1.83	1.59
Active survey data	65–79	11	135	8.15^*^	56,202	4,692,060	1.2	6.79
	80–90+	6	51	11.76^*^	43,407	753,528	5.76	2.04
	Total	17	183	9.29^*^	99,609	5,445,586	1.83	5.08

^*^Prevalence was estimated from other active survey study.

Using the NHI data from 2000, we identified the population without dementia and extracted their data through the period from 2000 to 2003. Overall, 99,609 new dementia cases were diagnosed from 2000 to 2003, leading to an incidence rate of 1.83%. The dementia incidence rate increased with age, doubling for increasing 5-year age bands, similar to the trend observed for prevalence. Among individuals 90 years and older, an ~19-fold increase in incidence was observed compared with those aged 65–69 years. Incidence was slightly higher among women (19.4%) than among men (17.3%).

The detection ratio was calculated to assess the extent of dementia awareness. The age-specific dementia detection ratios are presented in the last column of Table 1. Using the health insurance registry database, the overall dementia detection ratio was 1.53 in our study, and the detection ratio increased with age. In contrast, the detection rate for the community-based survey was larger than those estimated using the health insurance registry.

We also compared age-specific dementia incidence rates in four main geographical areas of Taiwan and found regional differences. Northern Taiwan has a higher incidence rate than Eastern Taiwan, and these findings suggest that urban areas have a higher incidence rate than rural areas. Table 2 shows the effects of age, sex, and geographical area on dementia risk based on the results of univariate and multivariate Poisson regression models. The results show that age, sex, and geographical area are associated with dementia risk.

**Table 2:** Effects of age, gender, geographic on the risk of incidence rate of dementia by Poisson regression model.

**Variable**	**Univariate**		**Multivariate**	

	**RR (95% CI)**	***P*** **-value**	**RR (95% CI)**	***P*** **-value**
Age		<0.0001		<0.0001
65–69	1.00		1.00	
70–74	1.02 (0.99, 1.04)		1.01 (0.99, 1.04)	
75–79	1.03 (1.01, 1.06)		1.03 (1.01, 1.05)	
80–84	1.05 (1.03, 1.08)		1.05 (1.03, 1.07)	
85–89	1.09 (1.06, 1.12)		1.09 (1.06, 1.12)	
90+	1.14 (1.11, 1.18)		1.14 (1.11, 1.18)	
Gender	1.01 (1.00, 1.02)	0.2003	1.01 (1.00, 1.03)	0.0450
Area		0.0057		0.0146
Central	1.00		1.00	
North	1.02 (1.00, 1.04)		1.01 (1.00, 1.04)	
South	1.00 (0.98, 1.01)		0.99 (0.98, 1.02)	
East	1.03 (0.99, 1.07)		1.02 (0.97, 1.06)	

Table 3 shows adjusted dementia detection rates. After adjusting for the geographical area, the detection ratio increased from 1.20 [95% confidence interval (CI): 1.15–1.24] for men in the 70–74-year-old group to 3.27 (95% CI: 3.13–3.41) for men in the ≥90-year-old group. A higher detection ratio of 1.45 (95% CI: 1.41–1.49) for men was observed for Northern Taiwan than for the three other geographical areas (Table 3). An opposite pattern was noted for women, with the detection ratio decreasing from 1.69 (95% CI: 1.63–1.74) for the 70–74-year-old group to 0.90 (95% CI: 0.86–0.94) for the ≥90-year-old group after adjusting for the geographical area. The detection rates among women were higher than those among men for all geographical areas.

**Table 3:** Adjusted detection ratios of dementia measured by passive survey.

**Variables**	**Regression coefficient (2.5–97.5%)**	**Adjusted detection ratios (2.5–97.5%)**
**Male**		
Intercept	0.197 (0.145, 0.248)	
**Age**		
65–69	Baseline	1.35 (1.29, 1.41)
70–74	−0.12 (−0.176, 0.063)	1.20 (1.15, 1.24)
75–79	−0.091 (−0.145, 0.037)	1.23 (1.19, 1.27)
80–84	0.141 (0.087, 0.195)	1.55 (1.51, 1.60)
85–89	0.36 (0.304, 0.417)	1.93 (1.86, 2.00)
90+	0.886 (0.823, 0.949)	3.27 (3.13, 3.41)
**Area**		
Central	Baseline	1.20 (1.17, 1.24)
North	0.186 (0.15, 0.222)	1.45 (1.41, 1.49)
South	0.096 (0.056, 0.136)	1.32 (1.29, 1.37)
East	−0.322 (−0.417, 0.228)	0.87 (0.80, 0.96)
**Female**		
Intercept	0.251 (0.2, 0.302)	
**Age**		
65–69	Baseline	1.47 (1.41, 1.54)
70–74	0.137 (0.082, 0.191)	1.69 (1.63, 1.74)
75–79	0.152 (0.1, 0.205)	1.71 (1.66, 1.76)
80–84	0.032 (−0.021, 0.085)	1.52 (1.47, 1.57)
85–89	−0.148 (−0.204, 0.091)	1.27 (1.22, 1.32)
90+	−0.492 (−0.557, 0.428)	0.90 (0.86, 0.94)
**Area**		
Central	Baseline	1.37 (1.32, 1.41)
North	0.211 (0.175, 0.247)	1.69 (1.64, 1.73)
South	0.025 (−0.015, 0.066)	1.40 (1.60, 1.44)
East	0.889 (0.807, 0.97)	3.32 (3.07, 3.59)

Table 4 shows the adjusted detection ratios for the passive (health insurance registry-based) and active (community-based) surveys. In the passive survey, detection rates increased from 1.45 (95% CI: 1.43–1.47) for the 65–79-year-old group to 1.64 (95% CI: 1.61–1.66) for the ≥80-year-old group. In the active survey, detection ratios increased from 4.23 (95% CI: 2.68–6.69) for the 65–79-year-old group to 4.77 (95% CI: 3.02–7.54) for the ≥80-year-old group.

**Table 4:** Adjusted detection ratios of dementia in comparison between passive and active survey.

**Variables**	**Regression coefficient (2.5–97.5%)**	**Adjusted detection ratios (2.5–97.5%)**
Intercept	0.252 (0.220, 0.283)	
Age 65–79 vs. age 80+	0.120 (0.100, 0.140)	
Active survey vs. passive survey	1.071 (0.614, 1.530)	
**Passive survey**		
Age 65–79		1.45 (1.43, 1.47)
Age 80+		1.64 (1.61, 1.66)
**Active survey**		
Age 65–79		4.23 (2.68, 6.69)
Age 80+		4.77 (3.02, 7.54)

The dementia detection rates estimated in our study were also compared with those reported by other community-based studies. Table 5 shows the dementia prevalence, incidence, and detection ratios in Taiwan as reported by other community-based studies. Generally speaking, these community-based surveys resulted in active dementia detection, with most reporting detection rates larger than 3, compared with the detection ratio of 1.59 estimated for the passive survey in the current study. Earlier studies, such as those conducted in Denmark and Sweden, reported lower detection rates. However, different diagnostic criteria over time may account for this disparity.

**Table 5:** Prevalence, incidence, and ratio of dementia in Taiwan and other community-based studies.

**References**	**Study period/ countries**	**Age range**	***N* for prevalence study**	***N* for incidence study**	**Area**	**Prevalence %**	**Incidence %**	**Detection ratio (prevalence/ incidence)**
Current (passive survey)	2000–2003/Taiwan	65+	1,921,308	1,921,308	Urban/rural	2.91	1.83	1.59
Current (active survey)	2013/Taiwan	65+	183	1,921,308	Rural	9.29	1.83	5.08
Chen et al. (9)	2004/Taiwan	65+	1,308	1,921,308	Urban/rural	10.55	1.83	5.77
Sun et al. (14)	2011–2013/Taiwan	65+	10,432	1,921,308	Urban/rural	8.14	1.83	4.45
Andersen et al. (17)	1985–1993/Denmark	65–84	3,299	3,086	Urban	7.10	2.95	2.40
Yoshitake et al. (18)	1985/Japan	65+	887	826	Sub-rural	6.7	1.8	3.72
Fratiglioni et al. (19)	1987/Sweden	75+	1,810	1,473	Urban	13.2	4.00	3.30
Fillenbaum et al. (20)	1986–1987/USA	68+/65+^*^	363	1,093	Urban/rural	7.10	1.93	3.68
Di Carlo et al. (21)	1992–1993/Italy	65–84	3,497	3,208	Urban/rural	8.26	1.33	6.20
Bermejo-Pareja et al. (22)	1994–1995/Spain	65+	5,278	3,891	Urban/rural	5.79	1.28	4.52
Chen et al. (23, 24)	2001–2003/China	65+	2,917	1,526	Rural	7.2	1.47	4.90

^*^Prevalence and incidence investigation for aged over 68 and over 65, respectively.

## Discussion

To the best of our knowledge, this is the first large-scale population-based study examining the dementia detection rate to simultaneously estimate the dementia prevalence and incidence within the same study. Our findings have significant implications for informing dementia etiology, patient behavior, healthcare infrastructure, and healthcare quality. The detection rate reflects the extent of dementia awareness, with a larger rate indicating enhanced awareness. Low awareness of dementia has been identified for routine healthcare, with active detection methods such as community-based surveys resulting in increased detection ratios than the passive detection approach utilized in the current healthcare system. The crude detection rate based on the health insurance registry data was 1.59, compared with 5.08 for the community-based study. Similar findings were observed for the age-adjusted detection ratios [passive: 1.45 (95% CI: 1.43–1.47) vs. active: 4.23 (95% CI: 2.68–6.69)] for individuals aged 65–79 years. A lack of dementia awareness among family members may result in delayed diagnosis and treatment. Several prospective longitudinal studies have demonstrated serious deficiencies in the ability of the healthcare system to recognize dementia, and most dementia remains unrecognized in the primary care setting. Persons with mild dementia are more likely to go unrecognized by both physicians and family members (over 90%) than persons with moderate to severe dementia (over 70%); however, those diagnosed during early disease stages are the most likely to benefit from treatment using currently available medications (15, 16). Our findings suggest that passive dementia detection results in underdiagnosis compared with active detection approaches, such as the community-based survey. Improved community-based dementia screening efforts should be considered to enhance dementia awareness and improve the early detection and treatment of dementia in Taiwan.

The statistical regression model used in the current study was developed to estimate the adjusted detection ratio to determine differences in dementia awareness and quality of care according to age, sex, and geographical area. This novel method takes into account associated covariates and is useful for estimating the degree to which disease detection is enhanced using active methods compared with passive methods.

We also compared the detection ratios estimated in the current study with those reported by other studies conducted in other countries (Table 5). The detection rates were 2.4 in Denmark (17), 3.72 in Japan (18), 3.30 in Sweden (19), 6.2 in Italy (20), 4.52 in Spain (21), 3.68 in the USA (22), and 4.90 in China (25). The detection rates reported for Japan and USA are similar to each other and were conducted during the same time frame. The studies conducted in Denmark and Sweden were also conducted in a similar time frame and reported lower detection ratios than the other studies, likely because these two studies were conducted earlier than the other studies, and they may not have included mild dementia cases. Dementia awareness and the application of approaches for early dementia detection were generally less common two decades ago. The lowest detection rate was reported at 2.4 among the Danish population for individuals aged between 65 and 84 years, whereas the Swedish study reported a higher detection rate (3.3) for an older population (aged over 75 years). The inconsistency in reported detection ratios may be due to differences in the underlying ages of the study cohorts and the use of different criteria for diagnosing dementia. The Danish study used the Diagnostic and Statistical Manual of Mental Disorders, Third Edition Revised (DMS-III-R) combined with the National Institute of Neurological and Communicative Diseases and Stroke/Alzheimer's Disease and Related Disorders Association (NINCDS-ADRDA) criteria to diagnose dementia, whereas the Swedish study only used the DMS-III-R criteria. The detection ratios reported by studies conducted after 1990 were considerably higher than those for earlier studies. Most high detection ratios were reported by studies based on active community-based surveys, reflecting a high awareness of the importance of early dementia detection.

Although the incidence rates did not differ substantially between men and women younger than 90 years, the prevalence in younger women was higher than that in younger men, which may account for higher detection ratios observed for younger women. The opposite results were noted for individuals older than 80 years, with a higher prevalence in men than in women, associated with a similar trend in detection ratios.

In the present study, we also estimated the age and sex-specific dementia incidence and prevalence rates in Taiwan. Women had an increased risk of dementia compared with men, which may be due to the longer life expectancy among women with dementia compared with men who are diagnosed with dementia at the same age.

Age is an independent risk factor for Alzheimer's disease (AD), and people aged 85 years or older are at the highest risk for AD. Jorm et al. documented the exponential rise in dementia diagnoses with age in several prevalence studies (26). In our study, starting at the age of 65 years, prevalence doubled every 5 years of age. However, our prevalence rate was considerably lower than those reported in other previous studies. Comparisons across studies should be taken with great caution as the majority of studies were based on an active community-based survey rather than population-based registry data, and the prevalence of dementia in the active community-based survey in our study increased to 9.3%, similar to the prevalence rates reported by previous community-based surveys conducted in Taiwan and other Western countries (Table 5). The awareness of dementia might also be lower in developing countries, and Libre et al. reported lower prevalence rates for developing countries, such as China and India, compared with more developed countries. Even with the active survey of prevalence, the underestimation of the true dementia prevalence remains a possibility because informant-based scores tended to be lower for heads of household and male participants in low- and middle-income countries (26). Although variations in incidence across countries exist, these disparities are smaller than those observed for prevalence, suggesting that dementia etiology is not substantially heterogeneous across different racial groups.

Only a few studies investigated the difference in the dementia detection ratio between active and passive screening. Nonetheless, we acknowledge that the present study has some limitations. The historical control in the present study was based on the NIH database, which collected the data from routine clinical practice. It was suggested that dementia diagnosis in clinical practice shows substantial variability in the detection methods and functional consequences of mild cognitive impairment (27). Therefore, the present study carries the risk of misclassification and ascertainment bias. Besides, the sample sizes between active and passive screening groups were substantially different; although we utilized adjustment models to account for this difference, sampling bias and errors cannot be excluded.

## Conclusion

The current study suggests that active screening of dementia can be beneficial and increase the detection ratio among the elderly. We demonstrated that the dementia detection ratio was higher for an active community-based survey than for a passive study based on health insurance registry data. Although these data can provide insights for healthcare stakeholders regarding the importance of increasing dementia awareness and the use of active surveillance in the elderly, our study should be supported by further evidence from prospectively collected data.

## IMPACT statement

This novel study has potential impacts on health policies. This study proposes that the detection ratio, defined as the ratio between prevalence and incidence, can provide new insights regarding awareness of early dementia detection in different medical care systems. The ratio can be derived using information available in either active or passive databases. This approach may encourage policymakers to routinely survey the potential dementia burden in communities and initiate early interventions in populations with low awareness.

## Data availability statement

The original contributions presented in the study are included in the article/supplementary material, further inquiries can be directed to the corresponding authors.

## Ethics statement

The studies involving human participants were reviewed and approved by China Medical University Hospital Institutional Review Board (No. CMUH104-REC2-060). Written informed consent for participation was not required for this study in accordance with the national legislation and the institutional requirements.

## Author contributions

Y-RL, YJ, and T-HW conceived the project and wrote the manuscript. AY, S-LP, and SC processed and analyzed the data. Y-RL, S-LP, and H-HC collected data. W-YL, W-CH, Y-CC, H-HC, and H-HL contributed to the data interpretation. All authors read and approved the final manuscript.
